# Chemokine-Directed Tumor Microenvironment Modulation in Cancer Immunotherapy

**DOI:** 10.3390/ijms22189804

**Published:** 2021-09-10

**Authors:** Pedro Bule, Sandra Isabel Aguiar, Frederico Aires-Da-Silva, Joana Nunes Ribeiro Dias

**Affiliations:** Centro de Investigação Interdisciplinar em Sanidade Animal, Faculdade de Medicina Veterinária, Universidade de Lisboa, 1300-477 Lisbon, Portugal; pedrobule@fmv.ulisboa.pt (P.B.); saguiar@fmv.ulisboa.pt (S.I.A.); fasilva@fmv.ulisboa.pt (F.A.-D.-S.)

**Keywords:** cancer, chemokine, chemokine receptor, immunotherapy, tumoral microenvironment

## Abstract

Chemokines are a large family of small chemotactic cytokines that coordinates immune cell trafficking. In cancer, they have a pivotal role in the migration pattern of immune cells into the tumor, thereby shaping the tumor microenvironment immune profile, often towards a pro-tumorigenic state. Furthermore, chemokines can directly target non-immune cells in the tumor microenvironment, including cancer, stromal and vascular endothelial cells. As such, chemokines participate in several cancer development processes such as angiogenesis, metastasis, cancer cell proliferation, stemness and invasiveness, and are therefore key determinants of disease progression, with a strong influence in patient prognosis and response to therapy. Due to their multifaceted role in the tumor immune response and tumor biology, the chemokine network has emerged as a potential immunotherapy target. Under the present review, we provide a general overview of chemokine effects on several tumoral processes, as well as a description of the currently available chemokine-directed therapies, highlighting their potential both as monotherapy or in combination with standard chemotherapy or other immunotherapies. Finally, we discuss the most critical challenges and prospects of developing targeted chemokines as therapeutic options.

## 1. Introduction

The immune system consists of a highly diverse network of cells tasked with maintaining homeostasis through the elimination of foreign pathogens and dysfunctional cells. In order to exert their function, immune cells need to come into contact with other cells. Therefore, their ability to migrate between and within organs in a context-specific manner is core to their function. This immune cell migration is guided by a set of small secreted chemotactic molecules called chemokines [1,2], which are a subfamily of cytokines responsible for immune cell trafficking and lymphoid tissue development [1,3,4]. Currently, there are 50 different chemokines reported, which can be grouped into four main classes, depending on the location of the first two cysteine (C) residues of their primary protein structure, namely the C, the CC, the CXC and the CX_3_C chemokines [2] (Table 1). All chemokines signal through binding to cognate heterotrimeric G protein-coupled receptors (GPCRs) of the rhodopsin-like family, found on the migratory cells [2] (Table 1). Each immune cell subset has a distinct chemokine receptor expression pattern, which makes them respond differentially to chemokines, migrating according to the special needs of each environment [5,6]. Nonetheless, there is a great deal of redundancy in the chemokine–chemokine receptor interaction. Out of the 19 canonical chemokine receptors, 14 are capable of recognising multiple ligands, while, in turn, chemokine ligands can bind to multiple receptors [3,7,8,9,10].

Aberrant expression of chemokine ligands or chemokine receptors has long been associated with dysfunctional lymphoid organ development and a defective or exacerbated immune response. Evidence now shows that chemokines are also key molecules for development and disease progression in the context of cancer [11]. The infiltration of immune cells into the tumor microenvironment (TME) is a determinant factor in cancer prognosis. Although chemokine signalling is crucial in recruiting immune cells with antitumor effects, such as CD8^+^ T cells, T_helper_ 1 (T_H_1) cells and natural killer (NK) cells, chemokine ligand secretion and chemokine receptor expression is often altered in the TME. This often leads to the recruitment of pro-tumorigenic immune cells such as myeloid-derived suppressor cells (MDSCs), tumor-associated neutrophils (TAN), tumor-associated macrophages (TAM) and regulatory T cells (T_reg_ cells). Proliferation of these cells as the disease progresses leads to the suppression of effector lymphocytes, and is associated with worse prognosis in patients with various types of cancer [12,13,14,15]. Additionally, chemokines can directly target non-immune cells in the TME, including tumor cells and vascular endothelial cells, which often display pathological chemokine receptor expression. Therefore, they can promote tumor cell proliferation, angiogenesis, cancer stemness, cancer invasiveness and metastasis. By acting directly and indirectly on the tumoral immune response, the chemokine system modulates tumor-immune and biological phenotypes and regulates cancer progression, which ultimately impacts therapy responses and patient clinical outcomes [16,17,18,19,20,21,22,23].

Due to the heterogeneous nature and undefined structure of tumors, the migration pattern of immune cells into the TME is unpredictable, as is the expression of chemokines and chemokines receptors. Therefore, TME composition can vary between tumors of the same type and even within the tumor itself [24]. Nonetheless, understanding the chemotactic environment of solid tumors, chemokine receptor expression in both immune and tumor cells and identifying chemokines that regulate immune cell recruitment into the TME is imperative in improving current immunotherapeutic interventions.

Here we review the role of chemokines in shaping TME composition and its effect in disease progression. We also discuss current efforts to target the chemokine–chemokine receptor axis for the treatment of several malignancies and the biggest challenges faced by this therapeutic approach.

## 2. The Roles of Chemokine Signalling in Cancer Development

**Figure 1:**
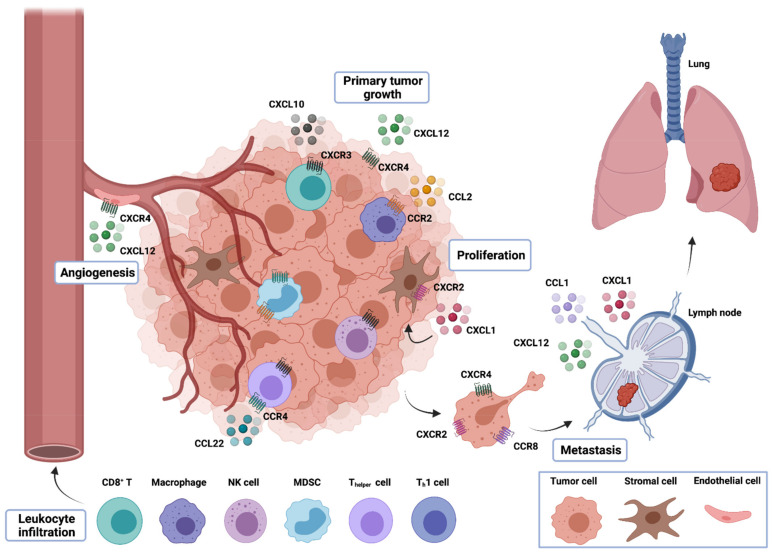
Multifaceted roles of chemokines in tumor development. Chemokines released by tumor cells, intratumor stromal cells, such as fibroblasts, and infiltrative leukocytes can recruit different immune cell types into the TME. The population of immune cells in the TME can interfere with the outcome of tumor development. While tumor- and stromal cell–derived chemokines can directly promote the growth, proliferation and survival of tumor cells, chemokines released by tumor cells, stromal cells and leukocytes can modulate the process of angiogenesis due to their angiogenic or angiostatic activity. Furthermore, chemokines produced within the tumor can induce the release of tumor-promoting growth factors that can act in a paracrine fashion to promote tumor growth. Finally, chemokines are also involved in the migration of tumor cells to distant sites for the development of metastasis.

As mentioned above, chemokine–chemokine receptor interactions are often altered upon oncogenic transformation, leading to dysregulation in these immune modulatory pathways, with consequences in multiple biologic mechanisms. Below, we discuss the role of chemokines in key cancer processes, such as immune evasion, tumor growth and progression, angiogenesis and metastasis (Figure 1).

### 2.1. Immune Evasion and Recruitment of Immunosuppressive Cells

**Figure 2:**
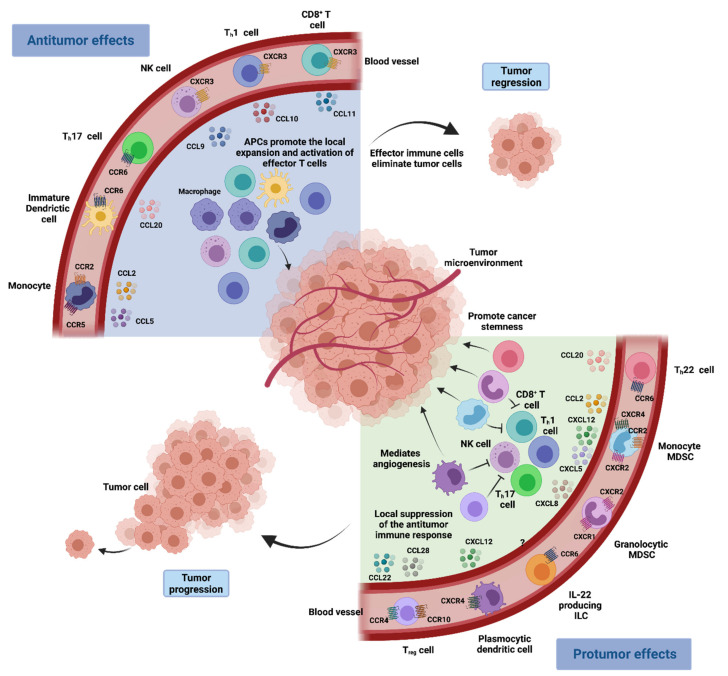
Chemokine network in the tumoral microenvironment immune response. Immune cells with antitumor effects such as CD8^+^ T cells, T_H_1 cells, polyfunctional T_H_17 cells and NK cells are attracted to the chemokine–chemokine receptor signalling pathways. CXCR3 and its ligands CXCL9 and CXCL10 have a main role in driving the migration of T_H_1 cells, CD8^+^ T cells and NK cells into the TME, while CCL20 signalling through CCR6 promotes the recruitment of T_H_17 cells. Furthermore, antigen presenting cells including macrophages and dendritic cells are also recruited into the TME, where they can activate and expand the local effector immune cells, promoting tumor regression. In turn, immune cell populations such as MDSCs, T_reg_ cells, T_H_22 cells, IL-22^+^ innate lymphoid cells (ILCs) and plasmocytic dendritic cells can promote tumor growth. These cells are recruited to the tumor bed in response to different chemokines that are expressed in the TME (the relevant receptors and ligands are shown). Immune cells with pro-tumorigenic actions may hinder antitumor immune responses and may also mediate and sustain cancer stemness and angiogenesis, resulting in cancer progression.

Immune evasion is a hallmark of carcinogenesis [25]. It is now widely accepted that cancer cells can successfully escape immune surveillance by overexpressing certain self-associated molecular patterns (SAMPs), often called “don’t eat me signals”. These can directly inhibit immune cell function through the activation of immune checkpoints or induce the differentiation of immunosuppressive cells that further restrain the activity of antitumoral cells [25]. Chemokines are critical in directing immune cell migration necessary to mount and deliver an effective antitumor immune response. Nonetheless, chemokine secretion is often altered in the TME, and an aberrant chemokine profile can facilitate the differentiation and infiltration of immunosuppressive pro-tumorigenic cells into the tumor, namely T_reg_ cells, MDSCs and TAMs [5] (Figure 2).

T_reg_ cells are a specialised subpopulation of CD4^+^ T cells that act to suppress the immune response, promoting self-tolerance and preventing exacerbated immune responses, thereby maintaining homeostasis [26,27]. Nonetheless, it has been shown that their infiltration into the TME often promotes immune tolerance and tumoral growth as they are capable of suppressing the activity of anti-tumoral T cells [28]. T_reg_ cells have higher expression of the chemokine receptor CCR4 than other CD4^+^ T cells [29]. Therefore, they are recruited into the TME in response to CCL22, a chemokine that is produced by TAMs and primary tumor cells. As such, elevated expression of CCL22 is correlated with lower spontaneous and therapy-induced T-cell antitumor immunity, leading to tumor growth and poor patient outcomes [18,29,30,31]. In addition to CCR4, T_reg_ cells can express other chemokine receptors capable of mediating their infiltration into the TME, such as CCR5 or CCR10, whose ligand CCL28 is found in hypoxic regions of the TME [32].

Macrophages are mainly recruited into the TME through the CCL2–CCR2 signalling pathway [33]. Tumoral expression of CCL2 correlates with the number of TAM in many tumors and is often associated with poor patient prognosis [34]. Like T_reg_ cells, TAMs can also inhibit tumor-associated antigens (TAA)-specific CD8^+^ T cell activation, which are capable of engaging tumor cells in an antigen-specific manner and drive antitumor immunity by secreting effector cytokines [35,36,37,38].

Myeloid-derived suppressor cells are a heterogeneous population of myeloid cells with immunosuppressive properties that includes monocytic and granulocytic cells [39]. It is believed that monocytic MDSCs can differentiate into TAMs at the tumor site and, much like TAMs, they can also be recruited into the TEM through CCL2–CCR2 signalling [40,41]. Because they share many features and possess similar immunosuppressive functions, monocytic MDSCs and TAMs are often used interchangeably in the context of cancer [4]. The immune-suppressive effects of monocytic MDSCs are relatively well-studied in mouse tumor models [39,42,43,44] and in patients with cancer [45,46,47]. They can directly suppress effector cells and recruit T_reg_ cells through the expression of CCL4. Once myeloid cells infiltrate the tumor, they can further produce the cognate ligand CCL2 and maintain or even augment monocyte trafficking into tumors [48,49].

Although the CCR2–CCL2 axis appears to be the main driver of TAM and MDSC recruitment, other chemokines have also been shown to contribute to the process. Increased CCL5 expression correlates with increased TAM infiltration and disease progression in breast cancer [50,51], while CCR5-expressing MDSCs have been shown to be more immunosuppressive than their CCR5^−^ counterparts [52]. Elevated CCL5 expression is also associated with disease progression in pancreatic [53], gastric [54] and ovarian cancer [55]. Other chemokines reported to induce monocyte recruitment to tumors are CCL7, CCL15, CXCL8 and CXCL12 [56]. Interestingly, a cascade involving CCL2 and CCL3 has been described, in which TAMs, derived from CCL2-recruited MDSCs, secrete CCL3, which further promotes macrophage retention in the tumor and tumor metastatic sites [40].

Plasmacytoid dendritic cells (pDCs) are a rare type of immune cells that are also capable of suppressing anti-tumoral immune response. These cells can be found in the human TME and have the ability to induce the development of IL-10-producing CD8^+^ T_reg_ cells that suppress DC activation of TAA-specific effector cells [57,58]. Tumor and stromal cells produce CXCL12, which is the ligand of chemokine receptor CXCR4, expressed by pDCs [59,60,61]. Therefore, CXCL12 is the key molecule for the recruitment of pDCs into the TME. Furthermore, CXCL12 also exerts a protective effect on plasmacytoid DCs, preventing them from undergoing apoptosis and prolonging their immunosuppressive action [58,59,60].

### 2.2. Tumor Growth and Progression

While normal cells maintain close regulation of cellular homeostasis by keeping a tight control on the synthesis and release of growth-promoting signals, these mechanisms are often impaired upon oncogenic transformation. As such, tumor cells commonly exhibit uncontrolled growth and proliferation due to a disruption of the regulatory mechanisms of growth factor production and signalling [25]. Several studies have suggested the involvement of the chemokine signalling system in tumor growth and progression, through different mechanisms [62]. The interaction between chemokine receptors expressed by cancer cells and their respective ligands secreted by tumor-associated fibroblasts, tumor cells and TME-infiltrating immune cells [63,64] can directly activate signalling pathways like Phosphoinositide 3-kinase (PI3K)/AKT and extracellular signal-regulated protein kinases 1 and 2 (ERK 1/2), leading to cancer cell proliferation [16,65,66]. These effects can be exacerbated by the pathologic overexpression of chemokine receptors on tumor cells and chemokine ligands secretion in the TME. Moreover, chemokines can sustain cancer cell survival by creating an imbalance between proapoptotic and antiapoptotic proteins in tumor cells (e.g., downregulation of Bcl-2 expression or inhibition of caspase-3 and caspase-9 activation), thereby avoiding tumor apoptosis [67,68].

However, the role of chemokines in tumor growth and progression is highly ambiguous as, parallel to their protumorigenic effects, these molecules actively participate in numerous inhibitory pathways that are crucial in preventing tumor progression [62]. In the first stages of oncogenesis, chemokines can hinder tumor growth and proliferation by mediating oncogene-induced senescence of tumor cells, which works as a natural mechanism against uncontrolled cell growth and malignant transformation [69,70]. The CXCL1/CXCR2 axis promotes oncogene-induced senescence through NF-κB signalling to restrain tumor growth. Nevertheless, in advanced stages of oncogenesis, senescent cells may also serve as a source of inflammation, mobilising MDSCs into the tumor site, contributing to an immunosuppressive microenvironment, and fostering tumoral growth [70,71].

Chemokine recruitment of certain immune cells can also contribute to tumorigenesis. The interleukin-22-secreting T_helper_ cells (T_H_22) are a subpopulation of immune cells often found in the TME, which have been shown to support tumorigenesis through several pathways, particularly in colon cancer. They express chemokine receptor CCR6 and migrate towards ligand CCL20, present in the TME, where they are able to increase cancer stemness and tumorigenic potential through cytokine expression [72].

### 2.3. Angiogenesis

Angiogenesis, defined as the formation of new blood vessels from pre-existing ones, is a crucial phenomenon for tumor growth and progression. Due to their characteristic rapid proliferation, tumor cells have increased demands in oxygen and nutrients as well as metabolic waste removal, which require accelerated neovascularisation to be met [25]. Chemokines and their respective receptors have been suggested as key regulators of tumor vasculature, possessing a binary role in tumor angiogenesis [73].

Based on the presence of the ELR (Glu-Leu-Arg) motif at the N-terminus, CXC chemokines can be divided into two groups: ELR^+^ chemokines and ELR− chemokines. Overall, ELR^+^ CXC chemokines, including CXCL1, CXCL2, CXCL3, CXCL5, CXCL6, CXCL7 and CXCL8 that act through the activation of CXCR1 and CXCR2, have angiogenic effects. On the contrary, ELR^−^ CXC chemokines such as CXCL4, CXCL9, CXCL10, CXCL11 and CXCL14 have been proposed as angiogenesis inhibitors. Still, this division is not absolute as CXCL12, an ELR^−^ chemokine, has been considered the most potent angiogenic chemokine [11,74,75,76].

Chemokines can work as tumor angiogenesis mediators by directly interacting with chemokine receptors on endothelial cells, which results in improved migration and proliferation and endothelial cell survival [73]. Additionally, chemokines may act indirectly by promoting the recruitment of leukocytes that produce angiogenic factors in the TME, enhancing angiogenesis [77]. TANs are the most notorious of the pro-angiogenic cells. Evidence suggests that TAN can differentiate to either exhibit antitumor or protumor features, but in most cases, the latter seems to prevail [78]. Neutrophils express receptors CXCR1 and CXCR2 in similar quantities, which guide them towards ligand CXCL8, as well as alternate ligands CXCL1, CXCL2, CXCL5 and CXCL6 [79]. Many of these ligands are overexpressed in several types of cancer, which correlates with high neutrophil infiltration, increased angiogenesis and poor prognosis [79,80,81,82].

Chemokines can also cooperate with other angiogenic promoters, such as the vascular endothelial growth factor (VEGF). The upregulation of VEGF expression induced by CXCL8 and CXCL12 results in a positive feedback effect in which VEGF further stimulates the production of angiogenic chemokines [73].

On the other hand, chemokines also possess inhibitory activity on tumor angiogenesis and endothelial cell proliferation. For example, CXCL4 and CXCL10 are chemokines with angiostatic properties that include the inhibition of angiogenesis induced by fibroblast growth factor and VEGF and the arrest of endothelial cell chemotaxis and proliferation. Furthermore, the interaction of CXCL9, CXCL10 and CXCL11 with CXCR3-expressing immune cells may recruit cells with angiostatic functions [74,83].

### 2.4. Metastasis

Metastasis is a complex process of malignant tumor dissemination from the primary tumor site to distant sites of the body, and remains a major cause of cancer-associated deaths [84]. Tumor metastasis involves a multi-step process known as the invasion–metastasis cascade. This order of events comprises the local invasion of primary cancer cells into surrounding tissues; intravasation of these cells into the bloodstream or lymphatic system and survival during circulation; arrest and extravasation through vascular walls into the parenchyma of distant tissues; formation of micrometastatic colonies in this parenchyma; and the subsequent proliferation of microscopic colonies into clinically detectable metastatic lesions, a phenomenon termed colonisation [85]. Numerous studies have attested the pivotal role that the chemokine system plays in metastasis. Indeed, it has been reported that chemokine receptor expression on cancer cells can define their secondary destination. Therefore, the production of specific chemokines by these metastatic sites can promote the migration of circulating cancer cells into a “premetastatic niche”, which presents a favourable environment for the growth of metastatic cells [73,86]. Multiple chemokines and chemokine receptors have been implicated in metastasis; however, the CXCL12/CXCR4 axis represents a critical actor of this phenomenon. Its involvement in tumor metastasis has been proven in different tumors, in which CXCR4 expression promotes the migration and metastasis of tumor cells into tissues with increased levels of CXCL12 [11,87]. Other examples of chemokines involved in cancer metastasis are CCR7, which mediates the migration of cancer cells to lymphatic organs through interplay with CCL19 and CCL21 ligands secreted in the metastatic site [86,88]; CCL28 expression, a ligand for CCR3/CCR10 that has been correlated with breast cancer growth and metastasis dissemination [89]; CCR10/CCL27 signalling supports the adhesion and survival of melanoma tumor cells during metastatic spread [90] and the CXCR5/CXCL13 interaction seems to support bone metastases in prostate cancer [91].

## 3. Chemokines in Cancer Therapy

The emergence of immunotherapies has revolutionised the field of oncology. By harnessing the host’s own immune system to target cancer cells, they have achieved unparalleled results, demonstrating the importance of the interaction between the human immune system and cancer. Although these therapies have proven clinically effective in a broad range of malignancies, clinical responses vary across patients and cancers. This disparity in clinical outcomes highlights the heterogeneity among different tumors and the highly intricate and regulated nature of their immune microenvironments. Due to the multifaceted roles that chemokines and their receptors play in cancer biology the chemokine system has been widely recognised as a source of potential new drug targets for cancer immunotherapy. Several targeting approaches have been pursued in preclinical studies (Table 2) and clinical trials (Table 3), which have culminated in the clinical approval of an anti-CCR4 antibody (Mogamulizumab) [92] and a CXCR4 antagonist (Plerixafor, AMD3100) [93] for haematological malignancies. Below, we summarise multiple efforts to target different chemokine receptor–ligand axis as therapeutic strategies for cancer, which are currently available or under development.

### 3.1. CCR1

CCR1 overexpression has been described in several types of cancer and is associated with increased immunosuppressive cell infiltration and metastasis [151,152,153]. Most of the therapeutic benefits of targeting CCR1 stem from reduced MDSC infiltration that culminates in the restraint of tumor growth and metastasis. A selective CCR1 antagonist, CCX721, was able to decrease tumor burden and osteolytic lesions in murine models of multiple myeloma (MM) bone disease, through the blockade of osteoclasts [94]. Similarly, halting CCL3, the ligand of CCR1, showed in vivo dual antitumor and antiosteolytic activity in MM [154]. Another work reported that the inhibition of CCR1 using the receptor antagonist BL5923 suppressed the recruitment of immature myeloid cells and reduced metastatic colonisation, significantly extending the survival of mice with hepatic metastasis of colon cancer [95]. The combination of a CCR1 antagonist, CCX9588, with an anti-PDL1 antibody has proven to be a promising therapeutic approach, as it resulted in synergistic antitumoral effects by inhibiting primary tumor growth and lung metastasis in an orthotopic breast cancer mouse model [96]. Recently, in a mouse model of ovarian cancer, the small molecule inhibitor UCB35625 was also able to decrease cell migration towards the omentum, a preferential metastasis site in this type of cancer [97]. Overall, these results suggested that targeting CCR1 can be a viable therapeutic strategy to limit dissemination and potentially slow disease progression.

### 3.2. CCR2, CCL2

The CCL2/CCR2 axis has been shown to be able to recruit immunosuppressive cells, such as MDSCs and metastasis-promoting monocytes, into the TME [33,155]. It is therefore a promising therapeutic target, whose blockade has resulted in antitumoral effects in several malignancies, by limiting the migration of monocytes with oncogenic and metastatic actions. Several works have focused on the therapeutic activity of CCR2 inhibitors on pancreatic cancers. Targeting TAMs through the inhibition of CCR2 signalling in an in vivo murine model using an oral CCR2 inhibitor, PF-04136309, increased chemotherapeutic efficacy, blocked metastasis and increased antitumor T-cell responses [98]. This same molecule in combination with Abraxane (nab-paclitaxel), a nanoparticle albumin-bound formulation of paclitaxel, and gemcitabine obtained favourable results in Phase Ib/II trial with metastatic pancreatic ductal adenocarcinoma (NCT02732938) [136]. Another phase II clinical trial evaluating this CCR2 inhibitor in combination with the conventional chemotherapy protocol FOLFIRINOX (FX), in patients with borderline resectable or locally advanced pancreatic ductal adenocarcinoma, confirmed the safety and tolerability of this therapy [137]. Notably, the inhibition of CCR2 using the small-molecule CCX872 improved the therapeutic benefits of anti-Programmed cell death protein 1 (PD-1)/Programmed cell death protein ligand 1 (PD-L1) immunotherapy in a syngeneic, orthotopic mouse model of pancreatic cancer [99]. A multi-centre trial in patients with locally advanced or metastatic, non-resectable pancreatic cancer evaluated the combination treatment of this inhibitor with FOLFIRINOX and reported an overall survival of 29% at 18 months with no safety issues [138]. A preclinical study in hepatocellular carcinoma assessing the blockade of CCL2/CCR2 with the CCR2 antagonist RDC018 revealed hindered tumor growth and metastasis, reduced postsurgical recurrence, and prolonged survival [100]. In turn, a natural CCR2 antagonist, 747, alone exhibited anticancer properties and potentiated the antitumor efficacy of a low dose of sorafenib in a mouse model of hepatocellular carcinoma [101]. These antitumoral effects were correlated with the elevation of CD8^+^ T cells via blocking CCR2-mediated recruitment of TAMs.

CCL2, the main ligand of receptor CCR2, is often overexpressed in many types of cancer and is associated with carcinogenesis. Although blocking CCL2 demonstrated preclinical antitumor activity by potentiating the effects of radiotherapy [156] and preventing metastasis [157], a phase 1 trial and phase 2 carlumab (CNTO 888), a human anti-CCL2 IgG1κ monoclonal antibody (mAb), in solid tumors (NCT00992186) and in metastatic prostate cancer (NCT00537368), respectively, failed to prove clinical benefit due to the inability of CNTO 888 to reduce CCL2 serum levels [139,140]. Curiously, Bonapace et al. (2014) revealed that despite the positive effects of an anti-CCL2 treatment on breast cancer metastases in mice, its interruption triggered an unwanted migration of monocytes into the metastatic site as well as an upsurge of IL-6 levels within the metastatic tissue. This culminated in increased blood vessel formation and metastases, which resulted in accelerated death [157]. These results prompted serious concerns when considering anti-CCL2 treatment.

### 3.3. CCR4

**Figure 3:**
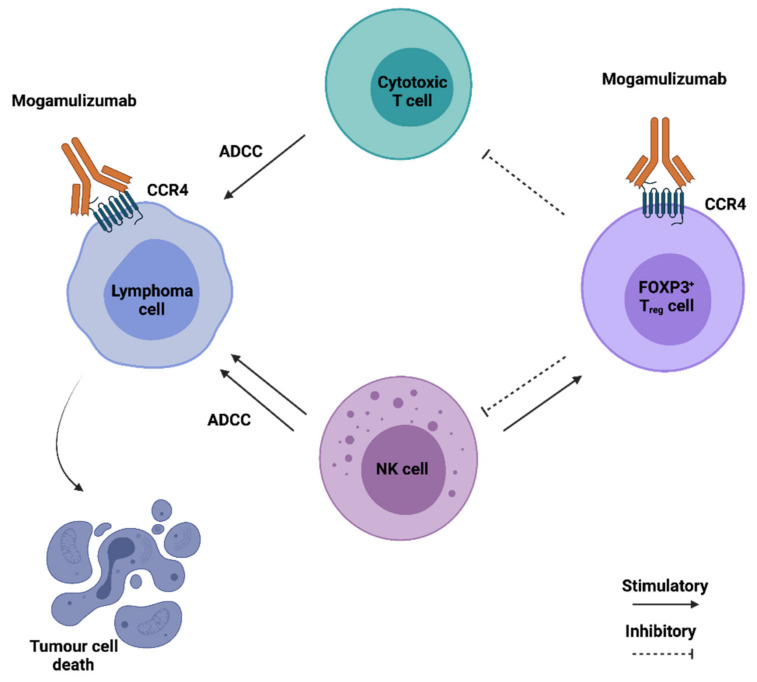
Anticancer therapeutic potential of Mogamulizumab (KW-0761). Mogamulizumab is shown in Figure 4. The antibody is prepared by glycoengineering and is defucosylated, enhancing the ADCC response promoted by cytotoxic T and NK cells. Elimination of CCR4^+^ T_reg_ cells further enhances the immune response of cytotoxic T cells against the tumor cells.

Besides being the main chemokine receptor in regulatory T cells, CCR4 is often overexpressed in several T cell malignancies. The anti-CCR4 antibody Mogamulizumab, initially developed to treat refractory Hodgkin lymphoma, is currently used in Japan for the treatment of relapsed adult T-cell leukaemia, and has successfully improved progression-free survival and quality of life in a phase III clinical trial of cutaneous T cell lymphoma [141,142]. It consists of a humanised mAb, with a defucosylated Fc region to enhance effector cell binding, capable of inducing malignant T cell elimination via antibody-dependent cellular cytotoxicity (ADCC) (Figure 3). The use of CCR4 blocking antibodies is also a promising strategy for the treatment of solid malignancies as they can cause T_reg_ cell depletion, therefore counteracting their immunosuppressive action in the TME. In an orthotopic mouse model of renal cell carcinoma, a fully human anti-CCR4 antibody was able to alter the phenotype of myeloid cells from pro to anti-tumorigenic, and increase the number of NK cells in the TME, thereby reducing tumor growth [102]. Nonetheless, a major side effect of T_reg_ cell depletion through the use of anti-CCR4 mAbs is the long-lasting effect on T_reg_ population. This can lead to auto-immunity or, in the case of patients previously subjected to allogenic bone marrow transplant, it can increase the risk of graft-versus-host disease [158]. Two independent clinical trials have revealed that Mogamulizumab can be safely used both alone or in combination with the anti PD-1 antibody Nivolumab for the treatment of advanced or metastatic solid malignancies [92,144]. Other CCR4-targeting strategies are currently under development. These include anti-CCR4 CAR-T cells, which have proven to be effective against several T-cell malignancies and small-molecule CCR4 antagonists capable of improving the efficacy of anti-cancer vaccines by preventing T_reg_ induction [103,104].

### 3.4. CCR5

CCR5 mediates physiologic functions of immune cells including T cells, macrophages, eosinophils, MDSCs, microglia and dendritic cells. Under physiologic conditions, CCR5 is expressed in immune cells, promoting their differentiation and migration to sites of inflammation. However, in many types of cancer, tumor epithelial cells can develop pathological expression of CCR5, induced upon oncogenic transformation, which allows them to hijack the migratory phenotype of immune cells, inducing a homing behaviour towards metastatic sites [105,109]. Furthermore, CCR5 is also involved in the mobilisation of myeloid cells with pro-tumoral activity TME, including T_reg_ cells, MDSCs and TAMs [159].

Some individuals carry a naturally occurring homozygous 32 bp deletion of the CCR5 coding region (*CCR5*Δ*32*), which imbues them with increased resistance to the human immunodeficiency virus. Since individuals who carry the *CCR5-*Δ*32* mutation are physiologically normal and CCR5 overexpression is found in various malignancies, recent interest has focused on retasking CCR5 antagonists developed for HIV treatment for cancer and cancer-related diseases.

The humanised monoclonal anti-CCR5 antibody, leronlimab, and the pyrimidine small-molecule CCR5 inhibitors, maraviroc and vicriviroc, have all shown promising results in several malignancies [105,106,108,110,160]. All three have shown the ability to block metastasis of human breast cancer xenografts in immunodeficient mice and to enhance cell killing by DNA-damaging chemotherapeutic agents [105,106,108]. Maraviroc and vicriviroc were also able to reduce cell metastasis in the whole body, bone and brain in a mice model of prostate cancer [107] while Maraviroc limited the accumulation of cancer-associated fibroblasts in a colorectal cancer model, leading to reduced tumor growth [110] and suppressed cell growth in an acute lymphoblastic leukaemia model [109]. Other CCR5-neutralising molecules, including the non-peptide antagonist TAK-779 and mouse anti-CCR5, have also shown promising results in pre-clinical models of pancreatic cancer and melanoma, by restricting the migration of MDSCs and T_reg_ cells into the TME [111,112]. Targeting CCL5, the primary ligand of CCR5, through bone-marrow gene silencing in combination with maraviroc administration has also led to a strong reduction in immunosuppressive myeloid cells and augmented antitumor immunity in a breast tumor mode [160].

Importantly, maraviroc has shown promising results in a clinical trial (MARACON), reducing cell growth in colorectal cancer patients that were refractory to standard chemotherapy [145], while two other clinical trials evaluating the combined PD-1 inhibition (Pembrolizumab) with Maraviroc or Vicriviroc, respectively, for the treatment of refractory MSS, revealed prolonged disease stabilisation and a higher survival rate than expected [143,161]. Additional clinical trials with CCR5^+^ metastatic cancer patients are currently underway to evaluate the combination of either a CCR5 antagonist with a biologic, or of leronlimab with a conventional chemotherapeutic agent [162].

### 3.5. CCR7

Much like CCR5, CCR7 is a chemokine receptor present in certain subsets of immune cells that can be pathologically expressed by tumor cells. This increases their homing behaviour and drives tumor growth and metastasis, particularly towards lymphatic organs, where the two ligands of CCR7, CCL19 and CCL21, are constitutively expressed [86,163]. Furthermore, certain tumors are capable of creating a CCL21-rich microenvironment, which correlates with high infiltration of T_reg_ cells and MDSCs [164].

CCR7 neutralisation therapy has shown promising results in a number of pre-clinical models.

The silencing of CCR7 gene expression through siRNA or miRNA led to decreased metastasis and tumor growth in models of prostate, breast and colorectal cancer [114,115,116]. Anti-CCR7 mAbs have shown the ability to induce tumor cell death and decrease or avoid central nervous system disease in a T-cell prolymphocytic leukaemia xenograft mice model [113], while single-chain anti-CCR7 antibodies successfully blocked the passage of T-cell acute lymphoblastic leukaemia cells through a blood–brain barrier in vitro model [165].

### 3.6. CXCR2

The CXCR2–CXCLs axis is a crucial chemotactic factor for the recruitment of immune suppressive myeloid cells to lesions in various inflammatory diseases and cancer. Increased expression of CXCR2 and CXCR2 ligands, both the main ligand CCL8 and alternative ligands, has been observed in many types of tumors and it seems to be related to the chemotherapeutic resistance observed in many cancers. The mechanisms by which the CXCR2–CXCLs axis promotes tumor progression are many, but the most notable is linked to the recruitment of neutrophils into the TME and the promotion of angiogenesis [166].

Neutralisation of CXCR2 has shown promising results in various preclinical cancer models, usually as part of combined therapies to circumvent chemotherapy resistance. CXCR2 deletion has led to decreased metastasis and improved response to paclitaxel in a mouse model of breast cancer [167]. In a melanoma model, the CXCR2 inhibitor Navarixin synergised with mitogen-activated protein kinase inhibition [117] whereas the inhibitor SB225002 improved the antiangiogenic therapy Sorafenib in an ovarian tumor model [118] and enhanced the therapeutic effect of cisplatin via the regulation of neutrophils’ infiltration in a lung cancer model [119]. The CXCR1 and CXCR2 inhibitor Reparixin was also able to improve tumor cell apoptosis and decrease tumor volume in a gastric cancer model, when in combination with 5-fluorouracil [120]. AZ13381758, a small-molecule inhibitor of CXCR2, was able to reduce metastasis and substantially improved life span in a pancreatic ductal adenocarcinoma model, when combined with gemcitabine [121], while in a prostate cancer model, CXCR2 inhibition by SB265610 was able to limit tumor growth by decreasing myeloid cell infiltration and enhancing Docetaxel-induced senescence [122].

Notably, seven CXCR2 inhibitors have been or are currently being investigated in several clinical trials, four of which for the treatment of metastatic malignancies. These include AZD5069/AZD9150 for the treatment of prostate cancer (Phase 2), squamous cell carcinoma of the head and neck (Phase 1b/2) and pancreatic ductal carcinoma (Phase 1b/2); Reparixin for the treatment of breast cancer (Phase 2); Navarixin for prostate and non-small cell cancer (Phase 2); and SX-682 for stage III and IV melanomas (Phase 1) [143,166].

Although the pharmacological intervention against CXCR2 has shown promising therapeutic benefits, some studies suggest that CXCR2 can potentially play a stage-dependent suppressive role in tumor development. As such, the CXCR2 blockade requires better understanding of the mechanisms underlying this chemokine axis in cancer biology [168].

### 3.7. CXCR4

**Figure 4:**
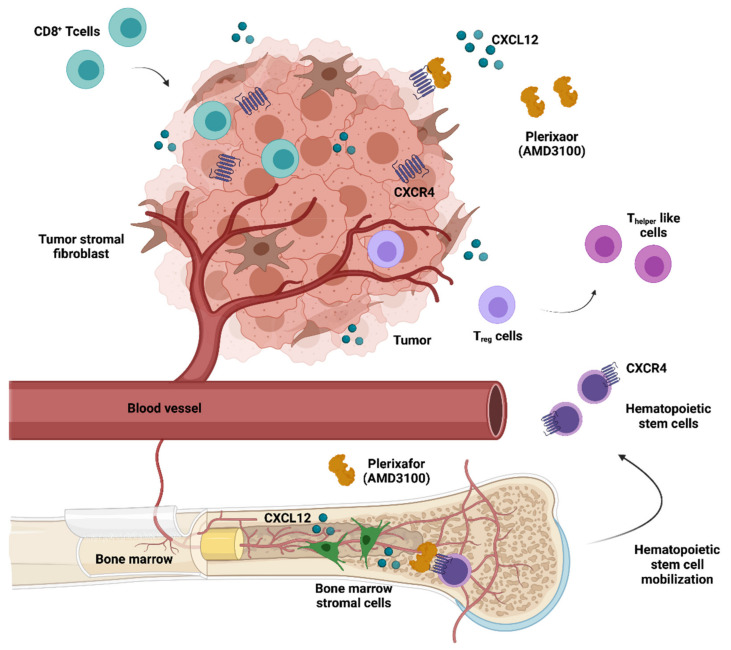
Anticancer therapeutic potential of AMD3100 (Plerixafor). Mozobil® (Plerixafor) was approved in combination with G-CSF to mobilise hematopoietic stem cells to the peripheral blood for collection and subsequent autologous transplantation in patients with NHL or MM. In addition, CXCR4 antagonists can disrupt the adhesive interactions between tumor cells and CXCL12-secreting stromal cells, mobilising them from the TME, and making the tumor cells more accessible to cytotoxic drugs. Furthermore, AMD3100 enhances the infiltration of CD8^+^ T cells, reduces immunosuppressive cells and converts Treg to Thelper-like cells in tumors.

Given the undisputed clinical relevance of CXCR4 regarding the growth and dissemination of a variety of malignancies, a multitude of CXCR4-directed peptidic and non-peptidic antagonists have been developed during the last decade [169,170]. The CXCR4–CXCL12 axis regulates the hematopoietic stem cell (HSC) niche. This has led to the approval of the CXCR4 antagonist plerixafor (AMD3100) as a hematopoietic precursor mobilisation agent, which releases HSCs into the peripheral blood for collection and subsequent autologous transplantation in Non-Hodgkin lymphoma (NHL) or MM patients [93]. Apart from constituting an HSC mobilising agent, both preclinical [123] and clinical studies [146] have suggested that AMD3100 and other CXCR4 antagonists exhibit anticancer activity, inhibiting tumor growth and metastasis as well as counteracting an immunosuppressive intratumoral microenvironment (Figure 4).

In the case of hematopoietic tumors, CXCR4 antagonists, such as AMD3100 and AMD3465, potentiated the clinical efficacy of conventional therapies by mediating the trafficking of tumor cells from the bone marrow milieu. Using a genetically defined murine model of acute myeloid leukaemia (AML), Nervi et al. (2009) showed that AMD3100 mobilised leukemic blasts into the peripheral circulation, sensitising them to the antitumoral effects of chemotherapy, thereby decreasing tumor burden and improving overall survival [123]. A phase I/II study in patients with relapsed AML presented consistent clinical data, reporting the correlation of in vivo evidence of disruption of the CXCR4/CXCL12 axis with encouraging rates of remission (NCT00512252) [146]. Similarly, a preclinical study in AML-bearing mice observed that the peptidic CXCR4 antagonist LY2510924 was capable of mobilising AML cells, had potent antileukemia activity and strongly synergised with cytotoxic chemotherapy [132]. The CXCR4-specific high-affinity antagonist BKT140 revealed potent in vivo anti-lymphoma properties that synergise with rituximab, by effectively targeting lymphoma cells in the bone marrow microenvironment and overcoming stroma-induced resistance to rituximab [126]. The humanised CXCR4 immunoglobulin G1 (IgG1) antibody PF-06747143 showed a strong antitumor effect in multiple hematologic tumor models including NHL, AML and MM. A phase I trial (NCT02954653) evaluating its safety and tolerability was conducted in acute myeloid lymphoma patients, but was unfortunately terminated due to sponsor prioritisation [147]. Recently, a phase Ib/II trial (NCT01359657) of another anti-CXCR4 antibody, ulocuplumab (BMS-936564), confirmed that blockade of the CXCR4–CXCL12 axis by this mAb is safe, with acceptable adverse effects, and leads to a high response rate in combination with lenalidomide and dexamethasone in patients with relapsed/refractory myeloma [148].

CXCR4 inhibitors have also been proved to have important anticancer potential in solid tumors. The expression of CXCR4 in human brain tumors and the potent anti-xenograft activity of the selective CXCR4 antagonists AMD 3100 and AMD 3465 position CXCR4 among the few validated targets for molecular therapy of malignant brain tumors [127,171]. Furthermore, the brain-penetrating CXCR4 antagonist, PRX177561, was capable of potentiating the antitumor effects of bevacizumab and sunitinib in preclinical models of human glioblastoma [128], while the combination of the antagonist POL5551 with an anti-VEGF agent resulted in inhibited tumoral growth and metastasis [129]. Several clinical trials are currently evaluating the clinical benefit of CXCR4 antagonists in glioblastoma patients. A phase I/II trial (NCT01977677) studied the side effects and best dose of plerixafor after temozolomide administration and radiation therapy. The first results revealed that plerixafor was safely escalated, with no dose-limiting toxicities observed, and appeared to inhibit CXCL4-mediated vasculogenesis in the post-RT period, enhancing the effects of radiation therapy [149]. Another phase I/II clinical trial evaluating the alternative inhibitor USL311 in combination with Lomustine in advanced solid tumors and relapsed/recurrent Glioblastoma Multiforme was recently terminated due to business reasons, not related to drug safety (NCT02765165).

Besides brain tumors, AMD3465 was also capable of preventing in vivo breast cancer growth and metastasis, while LY2510924, a novel cyclic peptide CXCR4 antagonist, exhibited antitumor activities in various solid tumor and metastatic breast cancer preclinical models [130,131]. LY2510924 was further tested in a phase I trial (NCT02737072) and proceeded to phase II trials after it was found clinically safe and well-tolerated in advanced solid cancers (colorectal, lung, breast and prostate). Promising preliminary results were obtained in a phase I trial (NCT01837095) with the CXCR4 antagonist balixafortide, both as a monotherapy as well as in combination with other agents, for HER2-negative metastatic breast cancer patients [150].

Remarkably, CXCR4 inhibition has also been shown to promote strong antitumor T cell responses. By hindering the interaction of CXCR4-positive tumor cells with CXCL12-producing fibroblasts, AMD3100 therapy in a pancreatic cancer model induced rapid T-cell accumulation among cancer cells and acted synergistically with anti-PD-L1 [133]. In turn, the modulation of intratumoral immunosuppression by AMD3100 improved the efficacy of the Mesothelin-Targeted vaccine VIC-008 in mesothelioma preclinical models, through the suppression of PD-1 expression in CD8^+^ T cells and the conversion of T_reg_ cells into T_helper_-like cells [134]. A CXCR4 blockade in an ovarian cancer preclinical model was also able to greatly increase T-cell–mediated antitumor immune responses, conferring a significant survival advantage to AMD3100-treated mice [124]. BPRCX807, a selective and potent CXCR4 antagonist, recently demonstrated promising in vitro and in vivo effects on hepatocellular carcinoma mouse models. This molecule significantly suppressed primary tumor growth, prevented distant metastasis/cell migration, reduced angiogenesis, and normalised the immunosuppressive TME by reducing TAM infiltration, reprogramming TAMs toward an immunostimulatory phenotype and promoting cytotoxic T cell infiltration into the TME [135]. Notably, the integrated immune effects of CXCR4 antagonists were also observed in human patients with microsatellite stable colorectal and pancreatic tumors treated with 1 week of continuous infusion of AMD3100 [125].

Certainly, a significant amount of effort has been dedicated to developing new CXCR4-targeting therapeutic strategies, which has led to very encouraging results in both preclinical studies and clinical trials. Based on the current literature, it is possible to envisage that targeting the CXCL12/CXCR4 axis in combination with immunotherapy and/or chemotherapy will become an important tool in oncological care.

## 4. Current Challenges and Future Perspectives

Recent achievements in chemokine-directed therapies highlight the tremendous potential that these immunotherapies have in the oncological setting. Their regulatory functions in both cancer cells and immune infiltrate cells make chemokine ligands and their receptors very powerful targets. Nevertheless, the development of TME-modulating therapies is incredibly challenging, and chemokine-directed therapies can be particularly difficult due to their broad expression and sometimes contradicting roles in tumor biology. Small-molecule inhibitors or antibodies targeting a chemokine or chemokine receptor are expected to have effects on all cells expressing those targets, both tumoral and immune, meaning that they can sometimes lead to unpredictable side effects. In the case of Mogamulizumab, which targets CCR4, this issue is more manageable because only a portion of T lymphocytes, including T_H_2, T_reg_ and T_helper_ 17 (T_H_17) cells, express CCR4. Considering the immunosuppressive response of T_reg_ and T_H_17 cells, their eradication could be beneficial in the context of cancer [147]. Even so, the long-lasting T_reg_ cell depletion induced by Mogamulizumab was partly associated with severe skin lesions and poses a threat to patients previously subjected to bone marrow transplants, as it can exacerbate graft-versus-host disease. This particular limiting factor of chemokine-directed therapy development is even more challenging when targeting chemokine receptors whose expression is not as differential as CCR4′s. In those cases, such as with CXCR4- or CCR7-directed therapies, a great proportion of leukocytes expresses the therapeutic targets, making the effects on the host’s immune response and the risks of severe immune-mediated adverse effects less predictable [172].

Another important shortcoming of these therapies is related to the complexity of the crosstalk between chemokines and the host immune system. Depending on tumor type, stage and immunological contexture, the inhibition of a specific chemokine–receptor axis may yield positive or deleterious effects on disease progression, acting as tumor suppressors or tumor promoters [173]. This can be exemplified by data gathered on CCR2^+^ tumor-infiltrating cells in several murine models: If the infiltrating cells are macrophages promoting the metastatic dissemination of tumor cells, the anti-CCR2 treatment may be effective; in contrast, if the infiltrating cells are CD8^+^ and γδ effector T-cells that improve immunosurveillance by enhancing T_H_1 responses, the treatment may be unfavourable [174].

Moreover, the likelihood of undesirable immune responses to chemokine-directed therapies can be potentiated when combining these therapies with standard-care treatments and, more importantly, immunotherapies, which has been the strategy of most clinical trials to date. The synergetic effects of these therapies with other agents have been widely described in the literature and have been considered generally positive. Nonetheless, while combinations of cancer therapeutics have the potential for enhanced efficacy, they also have the potential for increased toxicity.

Overall, the issues mentioned above raise important clinical translational concerns and underscore the need to determine the therapeutic windows, for each chemokine target and for different malignancies, in which the drug has an antitumoral effect, while not having a negative impact in the host’s immune system, in order to avoid potential side effects.

Notably, the lack of appropriate animal models reflecting the features and behaviour of human cancers also contributes to the difficulties in developing and translating novel chemokine-related target therapies [175]. Clinical translation of cancer immunotherapy relies on preclinical models to prioritise drug targets and investigate mechanisms of action, delivery approaches, treatment schedule, dose and safety [176]. Cancer-induced models often fail to mimic the heterogeneity and complexity of the interaction networks between the human immune cells and cancers and have continuously failed to correlate with clinical success rates [177,178,179]. To improve the success rate of immuno-oncology research and preclinical testing of immune-based anticancer therapies, preclinical models are being further refined to improve the tumoral immunogenicity by including humanised mouse models, genetically re-engineered mouse models, organoids and mammospheres derived from human tumor stem cell precursors, and ex vivo technology, as well as using alternative animal models more closely related to humans [180]. Dogs have been proposed as a powerful preclinical model of cancer immune therapeutics, serving as a bridge between laboratory animal models and humans [181,182]. By presenting intact immune systems that closely resemble the human immune system and by having analogous, spontaneous oncogenesis that elicits similar immune responses, pets can model key clinical outcomes such as efficacy, dose response and toxicity [183,184]. Recently, the profiles of chemokine and chemokine receptor gene expression in canine mammary carcinomas were associated with tumor behaviour in a way that was consistent with studies of human breast cancers [185]. In addition, T_reg_ cells migration mediated by CCR4 was associated with poor prognosis in dogs with spontaneous bladder cancer, mirroring a commonly observed feature in humans. Emerging evidence also suggests a potential role for chemokines in the biology of canine aggressive sarcomas, such as hemangiosarcoma and osteosarcoma [186,187]. In osteosarcoma-bearing dogs, zoledronate reduced CXCR4 expression within the primary tumor and decreased the circulating concentrations of CXCR4, demonstrating the potential of chemokine signalling modulation in the canine model [188]. Importantly, a canine clinical trial revealed that anti-CCR4 treatment with Mogamulizumab improved the survival rate while exhibiting a low rate of clinically relevant adverse effects, providing rationale for the translation of CCR4 blockade therapy to human patients with bladder cancer [189]. The same author reported that the CCR4 blockade led to clinical activity and prolonged survival in a canine model of advanced prostate cancer [190]. Altogether, these works confirmed the feasibility and clinical efficacy of these therapies in the veterinary setting and validated the potential of the canine model for the translation of chemokine-related immune therapies.

## 5. Concluding Remarks

The intricate nature of the interactions between chemokine receptors and their ligands, stemming from their concomitant expression on both immune cells and tumors (including tumor cells, stroma cells and/or tumor infiltrating cells) and the dichotomy of their elicited responses, highlights the double-edged potential for anti-chemokine immune therapeutics in cancer. To usher in a new generation of immuno-oncology therapeutic strategies based on chemokine modulation, a deep understanding of the tumoral microenvironment biology is urgently needed as well as better predictive clinical models. Due to their stage-dependent and person-to-person variability constraints, it is possible that these therapies may play an important role in personalised medicine, in which evidence derived from genetic, immune and proteomic profiling will inform therapeutic selection for each unique individual and their tumor.

Despite the challenges, a substantial number of chemokine receptor inhibitors, targeting different chemokine signalling pathways, are currently being evaluated in preclinical studies and clinical trials, showing promising results when used in combination with conventional chemotherapy or immune checkpoint therapy. As such, it is possible to predict that, in a not-so-distant future, chemokine receptor inhibitors will be used to modulate TME composition and optimise patients’ immune response, in order to overcome chemotherapy resistance.

## Figures and Tables

**Table 1:** Canonical chemokine receptors and their ligands, grouped by family.

Chemokine Family	GPCR	Ligands	Role/Expression of GPRC
CC	CCR1	CCL3, CCL4, CCL5, CCL7, CCL8, CCL13, CCL14, CCL15, CCL16, CCL23	Expressed on peripheral blood monocytes and memory T cells.
	CCR2	CCL2, CCL7, CCL8, CCL13, CCL16	It mediates monocyte chemotaxis and can be found on the surface of monocytes, basophils, B cells and activated memory T cells.
	CCR3	CCL4, CCL5, CCL7, CCL8, CCL11, CCL13, CCL15, CCL16, CCL23, CCL24, CCL26, CCL28	Expressed in eosinophils, basophils, T_H_2 cells, CD34^+^ hematopoietic progenitors, keratinocytes and mast cells present in several tissues. It is a co-receptor for HIV and plays a key role in allergic processes.
	CCR4	CCL3, CCL5, CCL17, CCL22	CCR4 is preferentially expressed on T_reg_ and T_H_2 cells. Expression can be transiently upregulated following TCR and CD28 engagement.
	CCR5	CCL2, CCL3, CCL4, CCL5, CCL8, CCL11, CCL13, CCL14, CCL16	It is the major co-receptor implicated in the susceptibility to HIV-1 infection. It is expressed on several cell types including peripheral blood-derived dendritic cells, CD34^+^ hematopoietic progenitor cells and memory T cells.
	CCR6	CCL20	Mainly expressed by immature dendritic cells and memory T cells, regulating their migration. Important for B lineage maturation and antigen driven B cell differentiation.
	CCR7	CCL19, CCL21	Expressed in lymphoid tissues and in activated B and T lymphocytes. Controls the migration of memory T cells to inflamed tissues and stimulates dendritic cell maturation.
	CCR8	CCL1, CCL4, CCL16, CCL17, CCL18	Preferentially expressed in the thymus. Regulates monocyte chemotaxis and thymic cell apoptosis. It contributes to the proper positioning of activated T cells within challenge sites and lymphoid tissues.
	CCR9	CCL25	Expressed in a range of hematopoietic cells, it is involved in chemokine-driven recirculation of leukocytes. It is differentially expressed by T lymphocytes of the small intestine and colon, suggesting it might be involved in the specialised immune response of the GI tract.
	CCR10	CCL27, CCL28	Mostly expressed by T_H_2 lymphocytes in the thymus.
	CCR11	CCL2, CCL8, CCL11, CCL13	Specifically binds the monocyte chemoattractant protein family of chemokines.
CXC	CXCR1	CXCL1, CXCL17	Mainly expressed on neutrophils, plays an important role in acute inflammation.
	CXCR2	CXCL1, CXCL2, CXCL3, CXCL5, CXCL6, CXCL7, CXCL8	Mainly expressed on neutrophils, plays an important role in acute inflammatory responses.
	CXCR3	CXCL4, CXCL9, CXCL10, CXCL11, CXCL13	Predominantly expressed on T lymphocytes. It is rapidly induced on naïve cells following activation and preferentially remains highly expressed on T_H_1 CD4^+^ T cells and effector CD8^+^ T cells
	CXCR4	CXCL12	Highly expressed in brain, heart, white blood cells, vascular endothelial cells and umbilical cord endothelial cells.
	CXCR5	CXCL13	Specifically expressed in Burkitt lymphoma and lymphatic tissues. Plays an essential role in B cell migration.
	CXCR6	CXCL16	Preferentially expressed on T_H_1 T cells. CXCR6 has been identified as a minor co-receptor for HIV-1 infection.
C	XCR1	XCL1, XCL2	Expressed on a subset of dendritic cells known to excel in antigen cross-presentation.
CX_3_C	CX_3_CR1	CX_3_CL1	Expressed in a variety of human tissues and cell lines where it mediates leukocyte migration and adhesion. Co-receptor for HIV-1 and HIV-2 infection.

**Table 2:** Pre-clinical studies of chemokine inhibition in cancer.

Target	Molecule	Cancer Type	References
CCR1	CCX721	Multiple myeloma	[94]
	BL5923	Colon cancer	[95]
	CCX9588 + PD-L1	Breast cancer	[96]
	UCB35625	Ovarian cancer	[97]
CCR2	PF-04136309	Pancreatic cancer	[98]
	CCX872 + anti-PD-1/PD-L1	Pancreatic cancer	[99]
	RDC018	Hepatocellular carcinoma	[100]
	747 + Sorafenib	Hepatocellular carcinoma	[101]
CCR4	Affi-5 (anti-CCR4 mab)	Renal cell carcinoma	[102]
	Anti-CCR4 CAR-T cells	T-cell malignancies	[103]
	AF399/420/1802 + vaccine + temsirolimus	Melanoma, lung and colon cancer	[104]
CCR5	Maraviroc and vicriviroc	Basal breast cancer	[105,106]
		Prostate cancer	[107]
	Leronlimab	Breast cancer	[108]
	Maraviroc	Acute lymphoblastic leukaemia	[109]
		Colorectal cancer	[110]
	TAK-779	Pancreatic cancer	[111]
	Anti-CCR5 mab (559921)	Melanoma	[112]
CCR7	Anti-CCR7 mab (150503)	T-cell prolymphocytic leukaemia	[113]
	siRNA	Metastatic colorectal cancer	[114]
		Metastatic prostate cancer	[115]
	miRNA	Breast cancer	[116]
CXCR2	Navarixin + MEK inhibitor	Melanoma	[117]
	SB225002 + Sorafenib	Ovarian cancer	[118]
	SB225002 + Cisplatin	Lung cancer	[119]
	Reparixin + 5-FU	Gastric Cancer	[120]
	AZ13381758 + Gemcitabine	Pancreatic ductal adenocarcinoma	[121]
	SB265610 + Docetaxel	Prostate Cancer	[122]
CXCR4	Plerixafor (AMD3100)	Acute myeloid leukaemia	[123]
		Ovarian cancer	[124]
		Colorectal and pancreatic cancer	[125]
	BKT140 + Rituximab	Lymphoma	[126]
	AMD 3100/AMD 3465	Brain cancer	[127]
	PRX177561 + bevacizumab + sunitinib	Glioblastoma	[128]
	POL5551 + anti-VEGF	Glioblastoma	[129]
	AMD3465	Breast cancer	[130]
	LY2510924	Metastatic breast cancer	[131]
		Acute myeloid leukaemia	[132]
	Plerixafor + anti-PD-L1	Pancreatic cancer	[133]
	Plerixafor + VIC-008	Mesothelioma	[134]
	BPRCX807	Hepatocellular carcinoma	[135]

**Table 3:** Clinical trials involving chemokine inhibitors for cancer treatment.

Target	Molecule	Cancer Type	Status	References	Identifier
CCR2	PF-04136309 + Abraxane + Gemcitabine	Metastatic pancreatic ductal adenocarcinoma	Phase Ib/II	[136]	NCT02732938
	PF-04136309 + FOLFIRINOX	Advanced pancreatic ductal adenocarcinoma	Phase II	[137]	NCT01413022
	CCX872 + FOLFIRINOX	Pancreatic adenocarcinoma	Phase Ib	[138]	NCT02345408
CCL2	Carlumab	Solid tumors	Phase I	[139]	NCT00537368
		Metastatic prostate cancer	Phase II	[140]	NCT00992186
CCR4	Mogamulizumab	Relapsed adult T cell leukaemia	Approved in Japan	[141]	
		Cutaneous T cell lymphoma	Phase III	[142]	NCT01728805
		Advanced solid tumors	Phase I/II	[92,143]	NCT02281409
	Mogamulizumab + Nivolumab	Advanced or metastatic solid tumors	Phase I	[144]	NCT02476123
CCR5	Maraviroc	Refractory colorectal cancer	Phase I	[145]	NCT01736813
	Maraviroc + Pembrolizumab	Refractory MSS-colorectal cancer.	Phase I	[143]	NCT03274804
	Maraviroc + Ipilmumab + Nivolumab	Metastatic colon and pancreatic cancer	Phase I	[143]	NCT04721301
	Vicriviroc + Pembrolizumab	Metastatic MSS-colorectal cancer.	Phase II	[143]	NCT03631407
	Leronlimab + Carboplatin	Metastatic triple-negative breast cancer	Phase Ib/II	[143]	NCT04313075
	Leronlimab	Solid tumors	Phase II	[143]	NCT04504942
CXCR2	AZD5069 + enzalutamide	Prostate cancer	Phase II	[143]	NCT03177187
	AZD5069/AZD9150 + MEDI4736	Head and Neck Squamous cell carcinoma	Phase Ib/II	[143]	NCT02499328
	AZD5069+ MEDI4736	Pancreatic ductal carcinoma	Phase Ib/II	[143]	NCT02583477
	Reparixin + Paclitaxel	Metastatic Triple-Negative Breast cancer	Phase II	[143]	NCT02370238
		HER2 Negative Breast Cancer	Phase I	[143]	NCT02001974
	Reparixin	Early breast cancer	Phase II	[143]	NCT01861054
	Navarixin + Pembrolizumab	NSCLC; Castration resistant prostate cancer; MSS- colorectal cancer	Phase II	[143]	NCT03473925
	SX-682 + Pembrolizumab	Stage III and IV melanomas	Phase I	[143]	NCT03161431
CXCR4	Plerixafor (AMD3100)	Acute myeloid leukaemia	Phase I/II	[146]	NCT00512252
	PF-06747143 (humanized mab)	Acute myeloid leukaemia	Phase I	[147]	NCT02954653
	Ulocuplumab + lenalidomide + dexamethasone	Relapsed/refractory myeloma	Phase Ib/II	[148]	NCT01359657
	Plerixafor + temozolomide + radiation	Brain cancer	Phase I/II	[149]	NCT01977677
	USL311 + Lomustine	Recurrent Glioblastoma Multiforme	Phase I/II	[143]	NCT02765165
	LY2510924LY	Solid cancers	Phase I/II	[143]	NCT02737072
	Balixafortide	Metastatic breast cancer	Phase I	[150]	NCT01837095

MSS—Microsatellite Stable; HER2—Receptor tyrosine-protein kinase erbB-2; NSCLC—Non-small cell lung cancer.
